# Effect of a Mobile Game–Based Intervention to Enhance Child Safety: Randomized Controlled Trial

**DOI:** 10.2196/51908

**Published:** 2024-02-14

**Authors:** Rosa S Wong, Keith T S Tung, Frederick K W Ho, Wilfred H S Wong, Chun Bong Chow, Ko Ling Chan, King Wa Fu, Patrick Ip

**Affiliations:** 1 Department of Special Education and Counselling The Education University of Hong Kong Hong Kong China (Hong Kong); 2 Department of Paediatrics and Adolescent Medicine The University of Hong Kong Hong Kong China (Hong Kong); 3 Institute of Health and Wellbeing University of Glasgow Glasgow United Kingdom; 4 Department of Applied Social Sciences The Hong Kong Polytechnic University Hong Kong China (Hong Kong); 5 Journalism and Media Studies Centre The University of Hong Kong Hong Kong China (Hong Kong)

**Keywords:** mobile game, injury prevention, safety education, unintentional injuries, gamification, game, games, gaming, safety, injury, injuries, gamify, gamified, child, children, youth, pediatric, pediatrics, danger, hazard, hazards, RCT, randomized, controlled trial, controlled trials, mobile phone

## Abstract

**Background:**

Evidence supports the effectiveness of serious games in health education, but little is known about their effects on the psychosocial well-being of children in the general population.

**Objective:**

This study aimed to investigate the potential of a mobile game–based safety education program in improving children’s safety and psychosocial outcomes.

**Methods:**

Safe City is a mobile roleplaying game specifically designed to educate children in Hong Kong about safety. This randomized controlled trial included 340 children in grades 4 through 6. Intervention arm participants (n=170) were instructed to play the Safe City mobile game for 4 weeks, whereas control arm participants (n=170) received a safety booklet. All participants completed a survey on safety knowledge and behaviors and psychosocial problems at baseline (T1), 1 month postintervention (T2), and 3 months postintervention (T3). Cumulative game scores and mini-game performance were analyzed as a proxy for the extent of exposure to the game. Outcome data were analyzed using 2-sample 2-tailed *t* tests to compare mean change from T1 to T2 and to T3 for intervention versus control arm participants. The association of game use with outcome changes postintervention was analyzed using generalized additive models.

**Results:**

No significant differences were found in mean changes between the intervention and control arms. However, use analyses showed that higher game scores were associated with improvements in safe behavior (*P*=.03) and internalizing problems (*P*=.01) at T3. Matching and Spot the Danger mini-game performance significantly predicted improvements in safety knowledge at T2 and T3.

**Conclusions:**

Analysis of use has shown that playing the Safe City mobile game can result in significant improvements in safety knowledge and reductions in unsafe behavior and internalizing problems. These findings provide evidence for the positive impact of serious games on psychological and social well-being, highlighting the potential of technology-driven interventions to assist children in learning about safety and preventing injuries.

**Trial Registration:**

ClinicalTrials.org NCT04096196; https://clinicaltrials.gov/show/NCT04096196

**International Registered Report Identifier (IRRID):**

RR2-10.2196/17756

## Introduction

Injuries remain a top contributor to mortality and disability worldwide, despite a decline on a global level [1]. Notably, children’s natural curiosity, energy, and desire for exploration can make them more susceptible to unintentional injuries [2]. Therefore, it is crucial to provide safety education to protect them from harm. Traditional teaching methods such as lectures, on-site demonstrations, and paper-based materials have shown success in preventing injuries, but they have limitations that could hinder their effectiveness in modern learning environments. For example, these methods may not provide enough stimulation or interaction with the material being taught, which can make it challenging for young students to feel connected with the subject matter and retain critical safety information. Recognizing the drawbacks of these conventional teaching methods, online technology is increasingly being used in health promotion because it offers a more captivating, personalized, and accessible approach to providing health education and information [3].

In recent years, there has been a growing trend of using games as an educational tool. Research has shown that this approach can improve students’ critical thinking skills, as well as increase their motivation and engagement in the learning process. [4]. Game-based learning can also stimulate curiosity and imagination, making the learning experience more enjoyable and interesting [5]. Serious games, which are computerized games developed for educational or training purposes, are more effective and efficient in achieving learning objectives compared to traditional teaching methods [6]. Meta-analyses have indicated that video game features such as engaging storylines and immediate feedback are beneficial for the promotion of health-related behaviors such as symptom management, physical activity, and diet [7,8]. On the other hand, only a limited number of research studies have used online game technology to enhance safety awareness and education among children. To our knowledge, only 1 trial study has used online game technology to enhance safety awareness and education among children. In the study, a mobile game app named iBsafe was created to reduce the risk of injuries associated with bicycles and dogs among kindergarten children in the United States [9]. Despite positive trial results [10], the intervention only lasted for 1 week and the follow-up assessment was done a week after the randomization. Therefore, the study only revealed short-term effects of the intervention. It remains unclear whether mobile game–based safety education programs can have a sustained impact on the safety knowledge and skills of young students.

Serious games serve not only as a means to enhance learning but are also frequently used as a tool in mental health interventions. Psychosocial problems are typically categorized into internalizing (eg, anxiety, depression, and social withdrawal) and externalizing problems (eg, hyperactivity, impulsivity, and rule violation) [11]. While the pooled evidence suggests a small yet significant association between increased screen time and a higher occurrence of internalizing and externalizing problems [12], trials provide evidence of the effectiveness of serious games, which are often based on cognitive behavioral therapy principles or incorporate elements of both exercise and gaming, in reducing symptoms of depression and anxiety [13,14]. A meta-analysis also reported that digital games created for therapeutic purposes were consistently associated with a reduction in negative emotional experiences among children and early adolescents [15]. Commercial video games, which are primarily designed for entertainment rather than therapy, were found to be as advantageous as clinical video games in producing feelings of joy, happiness, and positivity among older adults and patients [16]. Another meta-analysis found that the type of game (whether educational or therapeutic) did not moderate the impact of serious games on the mental health of children and adolescents, but the duration of the game and the length of each gaming session can have an impact [17]. Although the results yielded positive outcomes, notable discrepancies were observed, and evidence on externalizing problems remains limited, highlighting the necessity for further research to affirm the mental health consequences of serious games in the younger generation.

In Hong Kong, injuries pose a significant risk to childhood mortality and can result in long-term disabilities. A previous study revealed that between 2001 and 2012, 742,552 children and adolescents sought treatment for injuries at local public hospital emergency departments in Hong Kong. The majority of these injuries were unintentional, with home accidents (39%), sports-related incidents (18%), and road traffic accidents (4%) being the most common causes. The annual direct medical cost of these injuries amounted to US $29.4 million, with indirect costs reaching up to 2-3 times higher [18]. Among different age groups, domestic and sports injuries were among the top 3 leading causes of injury for children aged 5-9, 10-14, and 15-19 years, respectively. On the other hand, traffic injuries were more prevalent among younger children [18]. During the same study period, over 753 children lost their lives due to injuries, with traffic accidents accounting for the highest rate of unintentional injury-related deaths (0.69 deaths per 100,000 people) [18]. A study comparing childhood injuries in Hong Kong with 4 other countries (United States, Nigeria, Canada, and Greece) found that Hong Kong had the highest rate of falls (44%), similar to Greece, while transport injuries in Hong Kong (12%) were lower than in Nigeria (27%), but higher than in the other 3 countries (United States [6%], Canada [8%], and Greece [4%]) [19,20]. These findings underscore the importance of creating better tactics to educate children in Hong Kong about safety, particularly in the domains of home safety, sports safety, and traffic safety.

Accordingly, this study used a randomized controlled trial (RCT) design to examine the effectiveness of digital gaming methods compared to traditional booklet techniques in enhancing safety in the 3 specific domains of home safety, sports safety, and traffic safety, as well as in improving psychosocial outcomes among young students. In contrast to the traditional booklet method, the digital gaming methods included various mini-games such as Spot the Danger, Matching, multiple choice questions (MCQs), and Whac-A-Mole. These mini-games required players to engage in a learning loop where they applied, tested, and updated their safety knowledge. We hypothesized that students who received a mobile game–based safety education program would show significantly greater improvements in safety knowledge, safe behaviors, and internalizing problems compared to those who received a safety booklet. Additionally, we aimed to explore whether the level of improvement in postintervention outcomes was influenced by the total scores accumulated during gameplay. We hypothesized that higher cumulative scores would be associated with larger improvements in postintervention outcomes.

## Methods

### Study Design

**Figure 1:**
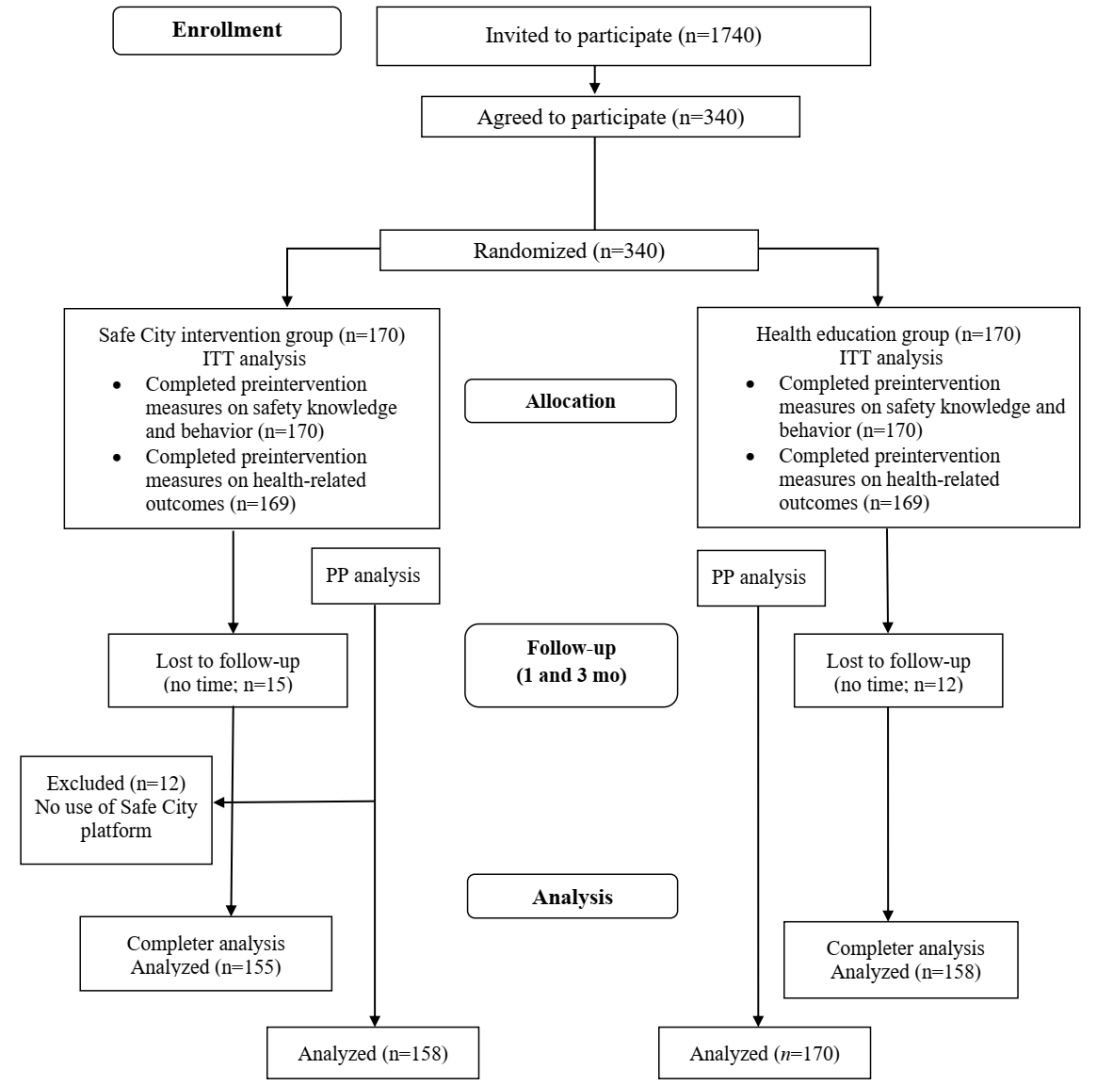
Study flowchart. ITT: intention-to-treat; PP: per-protocol.

We conducted an RCT to compare the effectiveness of a mobile game–based strategy and a traditional strategy to improve safety and psychosocial outcomes in a sample of 340 children aged 8-13 years in Hong Kong. The intervention was carried out over 30 days from enrollment to the trial end. Assessment was performed via a survey at baseline (T1), at 4 weeks postintervention (T2), and at 12 weeks postintervention (T3; Figure 1). A detailed study protocol has been published elsewhere [21]. The registered protocol for this study initially stated a larger sample size, but it was later reduced to 340 participants. The reason for the reduction was that the effect size cited in the registered protocol was based on preliminary results from a formative research study conducted with children and adolescents in the United Kingdom who likely had different learning preferences and experiences related to road safety compared to Hong Kong children [22].

### Sample Size Estimation

In 2020, the initial research in Hong Kong that used mobile apps and gamification methods to administer a physical activity intervention was published [23]. This study demonstrated a medium-to-large intervention effect on hyperactivity or inattention symptoms among healthy Hong Kong children aged 9-12 years, with an effect size of *d*=0.40 at a 6-month follow-up. Since hyperactivity or inattention symptoms are closely related to injuries and safety actions, and the results were derived from the local child population, we considered this result more suitable for estimating the sample size for this project. Based on the effect size of *d*=0.40, a minimum sample size of 200 (100 in each group) would be sufficient to achieve 80% statistical power at a 5% significance level.

### Recruitment and Participants

We recruited participants by distributing promotional leaflets through local mainstream primary schools across 7 districts in Hong Kong that had a significant prevalence of traffic, domestic, and sports injuries. These leaflets emphasized the appealing features of our newly developed educational mobile game. To be eligible for participation in this study, participants had to be in grade 4 to grade 6 and proficient in reading and writing traditional Chinese without the aid of an interpreter, as well as have access to a smartphone with internet connectivity. Exclusion criteria involved having neurodevelopmental disorders or suspected learning difficulties, as reported by teachers, parents, or caregivers.

### Procedure

Potential participants and their parents were directed to a study website with information about the project and web-based registration. After obtaining written consent for participation in this study, participants were granted access to a web-based screening survey that included questions about their demographic information and medical history. If participants did not meet any of the exclusion criteria, they were included in this study and invited to complete the T1 survey electronically. The included participants were then grouped into blocks, with each block consisting of 20 participants, based on the sequence of completion times for their T1 survey. Within each block, a data analyst who was not involved in other aspects of this study randomly assigned participants to either the intervention or control condition, using the R package *blockrand*, in a 1:1 ratio. Assignments were revealed to participants after they had completed the T1 survey. Participants were reassessed at T2 and T3 using the same survey that was administered at T1.

### Intervention

Participants assigned to the intervention arm played Safe City, a theoretically grounded roleplaying game that can work on both iOS and Android smartphones. The game’s design draws on social cognitive theory [24] and experiential gaming model [25], with input from experts, educators in child safety and injury prevention, as well as local children and their parents. Safe City is designed to promote safety knowledge and behavior by gamifying traditional safety education materials with digital and attractive features such as city scenes, character customization, and a scoring system. The game also uses story missions to enhance motivation for learning by instructing players to create and manage a fictional character (safety inspector) whose mission is to explore and eliminate potential injury hazards in the game’s world. The game world is divided into 5 zones, each dedicated to addressing safety risks in the 3 specific domains of home safety (2 zones), road safety (2 zones), and sport safety (1 zone). During the game process, learning happens in a continuous cycle. First, players identify potential injury risks and test their safety knowledge. Then, they apply this knowledge to ensure safety. Afterward, players reflect on the feedback received from the game and update their safety knowledge base accordingly [25].

Within each zone, there are 3 levels, each comprising of 5 components. In the first component, players are required to roleplay the correct way to cross a street by navigating through various checkpoints. In particular, the safety inspector (ie, the player) must adhere to standard street-crossing guidelines, such as avoiding jaywalking, while crossing a street in the game (Figure S1 in Multimedia Appendix 1). The remaining 4 components consist of 3 educational mini-games and 1 leisure-based mini-game, all of which are presented as checkpoints. The first educational mini-game (Spot the Danger) requires the safety inspector to perform a safety check and spot hazards in different areas within 35 seconds (Figure S2 in Multimedia Appendix 1); the second educational mini-game (Matching) requires the safety inspector to match safety signs with their corresponding description in 35 seconds (Figure S3 in Multimedia Appendix 1); and the third education mini-game is carried out in the format of MCQs, where the safety inspector is required to select a correct answer to the safety-related question within 35 seconds (Figure S4 in Multimedia Appendix 1). The educational mini-games award game points for correct answers and provide immediate feedback to the players by indicating which answer is correct after each question or gameplay. Lastly, the leisure-based mini-game adopts the Whac-A-Mole game design (Figure S5 in Multimedia Appendix 1). At the beginning of the gameplay, the safety inspector is presented with a list of safe and hazardous items. During the 35-second gameplay, the inspector is prompted to hit the items that randomly pop up from 9 holes. Whacking safe items earns points, but whacking dangerous items results in a loss of points. This experience of being rewarded for hitting safe items and penalized for hitting dangerous ones not only generates exhilaration but also motivates the player to continuously review and update their knowledge on safety. After the initial 35-second playtime, players have the option to replay any of the mini-games. However, to mitigate the risk of game addiction, every safety inspector begins with the same work energy level. A full energy level is indicated by 10, while no energy is represented by 0. When the energy runs out, players must wait for at least 2 hours to gain 1 energy step. In addition to the game zones, there is a designated rest area within the game known as the safety park. This area provides access to 3 other leisure-based mini-games that follow similar gameplay and rules as the Whac-A-Mole game but are presented in various styles. The points earned from participating in these mini-games determine the player’s position on the global scoreboard, which is another game feature designed to encourage use.

Intervention arm participants were instructed to play the Safe City mobile game with unlimited access for 30 days and asked not to share their own account and gameplay information with others until after the T3 assessment. Control participants had no access to the Safe City mobile game platform but received safety education in a traditional format, namely a booklet containing information about childhood injury hazards and safety measures, during this study period.

### Ethical Considerations

This study was approved by the ethics committee of the Institutional Review Board of the University of Hong Kong and Hospital Authority Hong Kong West Cluster (UW 19-028) and was registered with ClinicalTrials.gov (NCT04096196). Since the participants were aged younger than 18 years, their parents were required to provide separate written consent both for themselves and for their children to participate in this study. The data were deidentified before analysis and were securely stored in a password-protected file and on a password-protected computer. As an incentive, intervention players ranked in the top 20 were rewarded with a book coupon worth US $30.

### Survey Measures

#### Sociodemographic Questionnaire

A brief questionnaire was used to collect sociodemographic information including the participant’s gender and age. Parental educational attainment and household income were combined based on the factor loadings generated by the principal components analysis to create a composite family socioeconomic status index.

#### Safety Knowledge and Safe Behaviors

Safety knowledge and safe behaviors were measured using items adopted from the existing literature and questionnaires under the 3 contexts: home safety [26], road safety [22], and sports safety [27]. The wording of the items was modified and validated by local injury specialists to ensure their compliance with the safety regulations specific to the region. Participants provided ratings for their involvement in safe behavior on a 5-point Likert scale ranging from 0=never to 4=very often. For the safety knowledge items, a 4-point Likert scale was used, ranging from 1=strongly disagree to 4=strongly agree. The total scores, 1 for safety knowledge (Cronbach α=.61) and 1 for safe behavior (Cronbach α=.65) were calculated by averaging the relevant item scores and then transformed into a scale ranging from 0 to 100. Higher scores indicated a higher level of safety knowledge and safe behaviors.

#### Psychosocial Problems

Participants were asked to complete the psychosocial problem scales of the Strength and Difficulties Questionnaire, which included emotional symptoms, conduct problems, hyperactivity or inattention, and peer relationship problems [28]. The emotional symptoms and peer relationship problems scale scores were added up into an internalizing problem score (Cronbach α=.70), whereas the hyperactivity or inattention and conduct problems scale scores were added up into an externalizing problem score (Cronbach α=.79). Higher scores indicated more problems. The self-reported Strength and Difficulties Questionnaire version has been used on children between the ages of 8 and 13 years, and has demonstrated satisfactory psychometric properties [29].

#### Data Analysis

Before the analysis, we assessed the distribution, outliers, and missing data of all variables. We examined the skewness and kurtosis to evaluate the assumption of a normal distribution. Any potential outliers were eliminated if their values were ≥ 3 SDs from the mean of the group. Descriptive statistics were computed to characterize this study’s groups. Group differences were examined using chi-square tests for categorical variables or independent 2-tailed *t* tests for continuous variables. Changes in safety and psychosocial outcomes from T1 were compared between the 2 study groups at T2 and T3 using independent *t* tests. Sensitivity analyses were conducted to assess robustness. Intention-to-treat (ITT), per-protocol (PP), and completer analyses were conducted. The PP group included all control participants and those intervention participants who logged into the Safe City platform and accumulated a game score above zero. The completer group included all participants who completed the primary measures at T1-T3. In addition, generalized additive models (GAMs) were applied to explore the linear and nonlinear relationships between Safe City game use and participant outcomes adjusting for gender, age, baseline parameter value, family socioeconomic status index, random assignment, and school cluster. The effective degrees of freedom (edf) estimated from the GAM were used to reflect the degree of nonlinearity in the use-outcome relationship, with an edf of 1 indicating a linear relationship and a larger edf indicating a more complex, nonlinear relationship. Missing outcome data at T2 and T3 were imputed using the last value carried forward. R software (version 4.2.1; R Foundation for Statistical Computing) was used to perform all data analyses. The significance level was set at *P*<.05 (2-sided).

## Results

### Participant Characteristics

We recruited and randomly assigned 340 participants to either control (n=170, 50%) or intervention (n=170, 50%). Demographics are reported in Table 1. The sample included 166 (48.8%) males and 174 (51.2%) females, with a mean age of 10.71 years (SD 0.91 years). Among the participants, 52.6% (179/340) had a monthly household income below the median household income of US $3835 in Hong Kong. Additionally, approximately 34% (232/680) of parents had completed tertiary education, which was comparable to the population statistics in Hong Kong. Baseline demographics were similar between intervention and control groups, with the exception that fathers and mothers in the control group were slightly more likely to have completed tertiary education than those in the intervention group, although these differences were not statistically significant (*P*>.05).

**Table 1:** Baseline participant characteristics.

Characteristic			Intervention participants (n=170), n (%)		Control participants (n=170), n (%)		*P* value	
**Sex**								.19
	Male	89 (52.4)		77 (45.3)				
	Female	81 (47.6)		93 (54.7)				
Age (y), mean (SD)			10.70 (0.93)		10.72 (0.88)		.85	
**Monthly household income (US $)**								.33
	<3835	85 (50)		94 (53.3)				
	≥3835	85 (50)		76 (44.7)				
**Maternal education level**								.30
	Primary education or below	9 (5.3)		6 (3.5)				
	Secondary education	107 (62.9)		105 (61.8)				
	Tertiary education or above	54 (31.8)		56 (32.9)				
	Missing	0 (0)		3 (1.8)				
**Paternal education level**								.06
	Primary education or below	11 (6.5)		3 (1.8)				
	Secondary education	98 (57.6)		97 (57.1)				
	Tertiary education or above	59 (34.7)		63 (37.1)				
	Missing	2 (1.2)		7 (4.1)				

### Use

Of those randomized to the intervention condition, 100% (170/170) downloaded the Safe City app. In total, 96.4% (164/170) of participants logged into the platform at least once. Further, 92.9% (158/170) played at least 1 mini-game. The highest score accumulated over the 4-week intervention period was 14,598,100 and the lowest score was 4000, reflecting the wide-ranging use rates among the participants. On a scale of 1 to 30, the highest game level achieved was level 1 among 29.2% (38/158) of participants and level 15 or above among 8.2% (13/158) of participants.

### Effects of Intervention Participation

Table 2 presents the effects of participating in the Safe City game-based intervention on safety and psychosocial outcomes for ITT (n=340), PP (n=328), and the completer (n=325) analyses. At T1, both groups scored relatively high on safety domains (the average score was above 78 when the maximum score was 100) and relatively low on psychosocial problem domains (the average score was below 6 when the maximum score was 20). Overall, results showed that there were no meaningful variations between the groups in terms of mean change at T2 or T3, regardless of the method of analysis used. Additionally, the group × time interaction did not yield statistically significant results for any of the outcome measures.

**Table 2:** Baseline (T1) values and improvement of safety and psychosocial outcomes at 1 month (T2) and 3 months postintervention (T3).

Characteristic			Control (n=170)		ITT^a^ analysis					PP^b^ analysis					Completer analysis				
					Intervention (n=170)		*P* value		Intervention (n=158)			*P* value		Control (n=158)			Intervention (n=155)		*P* value
**Safe knowledge, mean (SD)**																			
	Baseline score^c^	78.11 (6.11)		78.11 (6.89)		N/A^d^		78.43 (6.65)			N/A		78.48 (6.04)			78.16 (6.83)		N/A	
	T1-T2^e^	0.12 (6.36)		–0.21 (6.17)		.62		–0.08 (6.14)			.77		0.13 (6.60)			–0.23 (6.47)		.62	
	T1-T3^e^	0.08 (6.13)		0.57 (6.46)		.47		0.67 (6.50)			.40		0.09 (6.36)			0.63 (6.76)		.47	
**Safe behavior, mean (SD)**																			
	Baseline score^c^	79.72 (10.23)		78.60 (11.00)		N/A		79.25 (10.72)			N/A		79.94 (10.26)			78.80 (10.90)		N/A	
	T1-T2^e^	0.75 (10.12)		0.95 (9.59)		.85		0.98 (9.52)			.83		0.80 (10.49)			1.04 (10.04)		.84	
	T1-T3^e^	0.80 (10.27)		1.43 (10.20)		.57		1.19 (9.87)			.73		0.86 (10.65)			1.57 (10.67)		.56	
**Internalizing problems^f^, mean (SD)**																			
	Baseline score^c^	4.80 (3.08)		4.72 (3.28)		N/A		4.64 (3.27)			N/A		4.80 (3.09)			4.75 (3.36)		N/A	
	T1-T2^g^	0.15 (2.71)		0.27 (2.98)		.69		0.23 (2.97)			.80		0.16 (2.82)			0.30 (3.12)		.68	
	T1-T3^g^	0.17 (3.00)		–0.03 (2.79)		.54		–0.01 (2.83)			.58		0.18 (3.12)			–0.03 (2.92)		.54	
**Externalizing problems^f^, mean (SD)**																			
	Baseline score^c^	5.95 (3.59)		5.79 (3.78)		N/A		5.65 (3.75)			N/A		5.85 (3.50)			5.89 (3.83)		N/A	
	T1-T2^g^	0.07 (2.71)		–0.11 (2.53)		.55		–0.08 (2.56)			.63		0.07 (2.81)			–0.12 (2.65)		.55	
	T1-T3^g^	–0.22 (3.03)		–0.36 (2.96)		.68		–0.29 (2.96)			.84		–0.24 (3.14)			–0.40 (3.10)		.66	

^a^ITT: intention-to-treat.

^b^PP: per-protocol.

^c^Mean domain score at baseline for participants in each study arm.

^d^N/A: not applicable.

^e^Positive values indicate a desirable change in scores.

^f^Missing data from 1 participant.

^g^Negative values indicate a desirable change in scores.

### Effects of Intervention Dosage

Table 3 presents the results of GAMs of safety and psychosocial outcome changes at T2 and T3 as a function of the Safe City game use. We found a significant and linear relationship between cumulative game scores and improvements in safe behaviors at T3 (*P*=.03), with higher game scores predicting greater improvements in safe behaviors (Figure S6B in Multimedia Appendix 1). The linear association can also be seen between cumulative game scores and improvements in internalizing problems at T3 (*P*=.01). The level of improvements in internalizing problems at T3 increased monotonically with an increase in game scores during the intervention period (Figure S6C in Multimedia Appendix 1). Regarding mini-games, we observed a significant and monotonic increase in safety knowledge improvements at T2 and T3 as the number of correct answers in Spot the Danger (Figure S8A in Multimedia Appendix 1) and Matching mini-games (Figure S9A in Multimedia Appendix 1) increased. Curvilinear associations were found between MCQ performance and improvements in safety knowledge at T2 (*P*=.008; Figure S7A in Multimedia Appendix 1). At T3, the relationship between MCQ performance and improvements in safety knowledge became linear and marginally significant (*P*=.053; Figure S7A in Multimedia Appendix 1). As the number of correct answers in MCQs increased, the improvements in internalizing problems at T3 became more apparent (*P*=.005; Figure S7C in Multimedia Appendix 1). For the Matching mini-games, the improvements in internalizing problems at T3 reached a plateau when more correct answers were achieved (*P*=.06; Figure S4C in Multimedia Appendix 1). No relationship was found between these game use parameters and changes in externalizing problems at T2 and T3 following the intervention.

**Table 3:** Effect of game use on improvement in safety and psychosocial outcomes at the 1 month (T2) and 3 months postintervention (T3).

Outcome		Cumulative game score		Multiple choice questions		Spot the Danger		Matching	
		edf^a^	*P* value^b^	edf	*P* value^b^	edf	*P* value^a^	edf	*P* value^a^
**Safe knowledge**									
	Improvement, T2	4.27	.35	5.11	.008	1	.008	1	.002
	Improvement, T3	1	.27	1	.05	1	.02	1	.03
**Safe behavior**									
	Improvement, T2	1	.07	1	.67	1	.58	1.12	.45
	Improvement, T3	1	.03	1	.48	1.41	.68	1	.53
**Internalizing problems**									
	Improvement, T2	3.07	.09	1	.09	1.17	.27	1	.12
	Improvement, T3	1	.01	1.12	.005	1	.20	1.66	.06
**Externalizing problems**									
	Improvement, T2	1	.72	1	>.99	1	.85	1	.42
	Improvement, T3	1	.51	1.17	.70	1	.83	1	.64

^a^edf: effective degrees of freedom.

^b^Adjusted for gender, age, baseline level, family socioeconomic status, random assignment, and school cluster.

## Discussion

### Principal Results

This RCT aimed to investigate the effectiveness of a mobile game–based safety education program in improving children’s safety and psychosocial outcomes. The results showed that participating in a 4-week Safe City game-based intervention did not lead to significant improvements in safety and psychosocial well-being at T2 and T3. However, we found evidence of significant effects of game use on changes in safety and psychosocial outcomes after the intervention. The notable enhancement in safe behavior and decrease in internalizing problems at T3 with increasing game scores (intervention dosage), indicates that the game is an effective and enjoyable tool for learning about safety. This, in turn, contributes to the improvement of safety and psychosocial well-being among the participants. Indeed, the analysis of use showed significant effects of the question types used in mini-games on the postintervention changes in safety knowledge and internalizing problems. Specifically, the Matching and Spot the Danger mini-games proved to be effective in promoting safety knowledge. On the other hand, the MCQ mini-game was found to be effective in reducing internalizing problems, although its effect on knowledge gains was not as strong as the other 2 types of mini-game.

### Comparison to Prior Work

In this study, it was observed that higher game scores during the intervention period were linked to a greater improvement in safe behavior at T3. The widely used Knowledge, Attitude, and Practices model in health research suggests that repeated acquisition of proper health-related knowledge leads to the formation and maintenance of healthy behavior [30]. While cumulative game scores did not have an impact on the improvement of safety knowledge postintervention, performance in educational mini-games was associated with changes in safety knowledge after the intervention. This finding may be attributed to the fact that the Safe City mobile game includes both educational mini-games and leisure mini-games, all of which award game points for successful attempts. While overall game scores reflect the level of engagement with the intervention, the educational mini-games specifically aim to directly modify or reinforce existing knowledge. By improving safety knowledge, individuals are more likely to adopt and maintain safe behavior, leading to an overall improvement in their safety practices at T3.

Although the results of the use-outcome analysis provide support for the efficacy of the Safe City mobile game as an educational tool to enhance safety and psychosocial well-being in children, it is important to note that the observed differences in mean changes between intervention and control participants were not statistically significant. Further, one possible explanation for this finding is that the participants, regardless of their assignment to the intervention or control group, already had a considerable amount of safety knowledge and compliance, as well as a low level of psychosocial problems, at the beginning of this study. The preexisting level of positive attributes may have limited their potential for improvement through the intervention. A previous study has suggested that demonstrating the effectiveness of an intervention can be challenging if participants’ scores on outcome assessments at the start of the study (ie, ceiling effects) are already within the desirable range [31]. Given the promising results regarding the use of the game, it is likely that the Safe City mobile game–based intervention will yield more favorable participation outcomes in scenarios where most participants have insufficient safety awareness and behaviors, or among younger participants who have not yet acquired adequate safety knowledge.

Another encouraging finding is that the intervention showed no effect on externalizing problems, such as conduct problems and symptoms of hyperactivity or inattention, regardless of participation in the intervention or the dosage received. On the other hand, we noticed that the positive effects of game use on internalizing problems were delayed. Specifically, we found that higher cumulative game scores were only associated with greater reductions in internalizing problems at T3. These findings are consistent with emerging research showing that playing video games may not be entirely detrimental to children’s mental health [32]. Previous reviews have highlighted the potential benefits of video games in promoting student engagement and motivation for learning. When used appropriately, video games can even serve as a powerful tool for enhancing cognition in children with mental disorders [33]. Furthermore, studies have demonstrated that various types of video games, including roleplaying games and even short gameplay sessions as brief as 1 or 5 minutes, can help reduce stress and anxiety [34]. Nevertheless, it is important to note that children with self-regulation difficulties, such as attention-deficit/hyperactivity disorder, may be more prone to problematic gaming behavior [35]. Hence, it is crucial for future research to explore the potential benefits of mobile game–based education interventions for both typically developing children and those with special education needs. Understanding how these interventions can effectively support the learning and development of all children, regardless of their individual circumstances, is of utmost importance.

Another notable finding is the differential effects of the types of questions used in mini-games on the postintervention changes in safety and psychosocial outcomes. In this study, the Matching and Spot the Danger performance showed a linear relationship with the postintervention increase in safety knowledge. On the other hand, the MCQ performance exhibited a nonlinear relationship with the improvements in safety knowledge and internalizing problems after the intervention. This distinction could be attributed to the higher involvement of guessing in MCQs compared to the other 2 types of questions. Previous studies have shown that matching items are better at differentiating well-prepared students from marginal students and are less susceptible to cueing issues compared to MCQs [36]. Therefore, it is plausible to speculate that participants who had a limited number of correct answers in MCQs may have relied more on guessing. This could have led to a wider range of knowledge improvement among participants at T2, which subsequently declined at T3.

### Limitations and Strengths

One of the major strengths of this study was collecting game use data as a proxy for intervention dosage. However, some limitations need to be taken into consideration when interpreting the findings. Firstly, 93% (158/170 participants) engaged in playing the Safe City mini-games at least once, but there was a significant variation in use rates, with game scores ranging from 4000 to 14,598,100. This variability poses challenges in obtaining an unbiased estimation of the intervention’s effects. Secondly, the sample had a minimal number of participants with insufficient safety knowledge or inadequate safety practices. Due to the limited statistical power, we were unable to investigate whether this specific subgroup would show more positive responses to the Safe City mobile game–based intervention. Lastly, the outcome measures relied on self-reports provided by the participants. This introduces the potential for social desirability bias, as individuals may provide responses that align with societal expectations rather than their true experiences or behaviors.

### Conclusions

This study contributes to the growing body of research suggesting that serious games, especially when engaged with at high levels, can effectively yield immediate and long-term improvements in knowledge and psychosocial outcomes among typically developing children. Additionally, the findings support the investigation of gamification as a viable approach to health education. Furthermore, this study underscores the importance of integrating traditional and digital teaching methods in education to maximize the benefits of digital tools. It may be necessary to develop question items in various styles, such as matching or MCQs, to attain different outcome objectives, which would require further evaluation.

## Appendix

Multimedia Appendix 1 Additional figures and tables.

Multimedia Appendix 2 CONSORT-eHEALTH checklist (V 1.6.1).

## Abbreviations

edf: effective degrees of freedom
GAM: generalized additive model
ITT: intention-to-treat
MCQ: multiple choice question
PP: per-protocol
RCT: randomized controlled trial

## Data Availability

The data that support the findings of this study are available on request from the corresponding author.
