# Supplementary material for: Effect of a Mobile Game–Based Intervention to Enhance Child Safety: Randomized Controlled Trial
Source: J Med Internet Res. 2024 Feb 14;26:e51908. doi: 10.2196/51908 (PMC10902767; doi:10.2196/51908)
Supplement: Multimedia Appendix 1 [file jmir_v26i1e51908_app1.pdf]

Figure S1. Safe City mobile game screenshot – road crossing

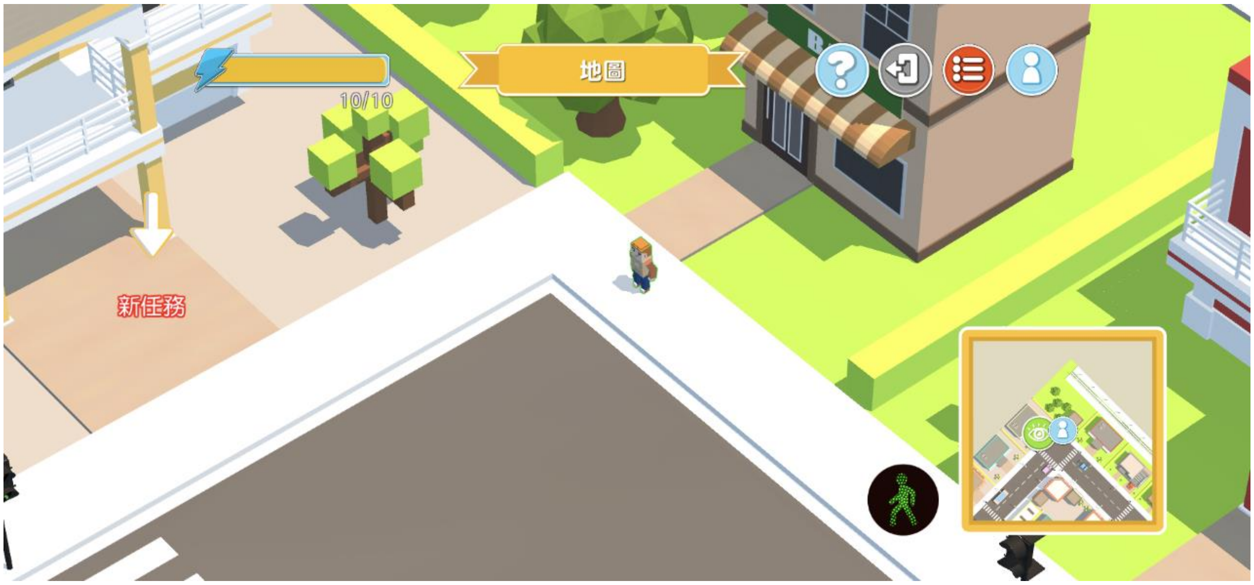

Figure S2. Safe City mobile game screenshot - Spot the Danger

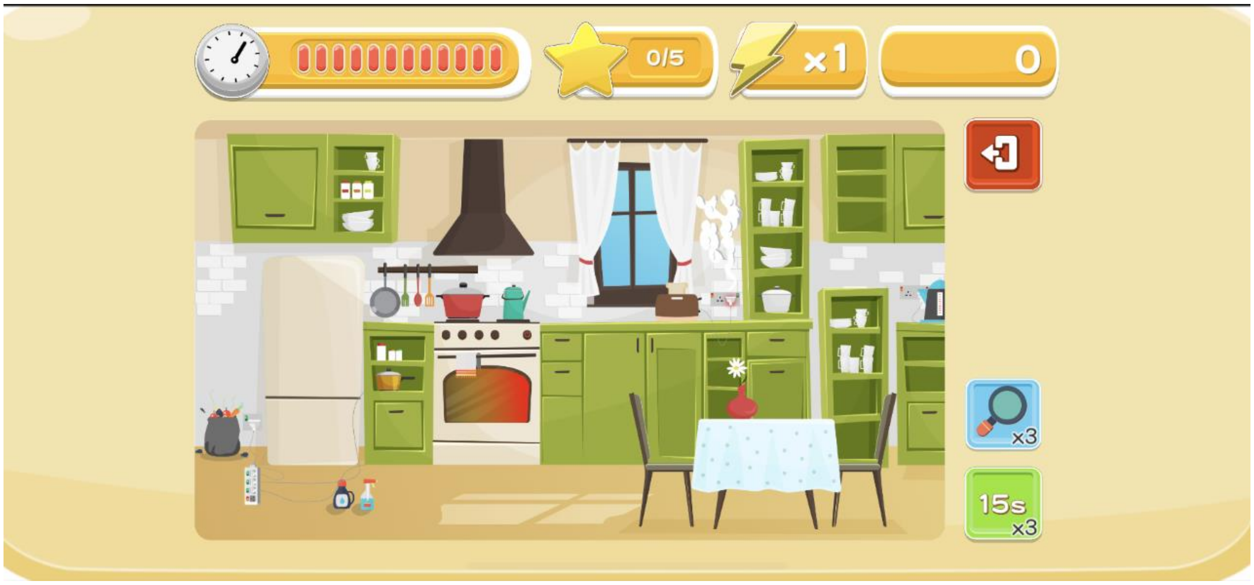

Figure S3. Safe City mobile game screenshot – Matching

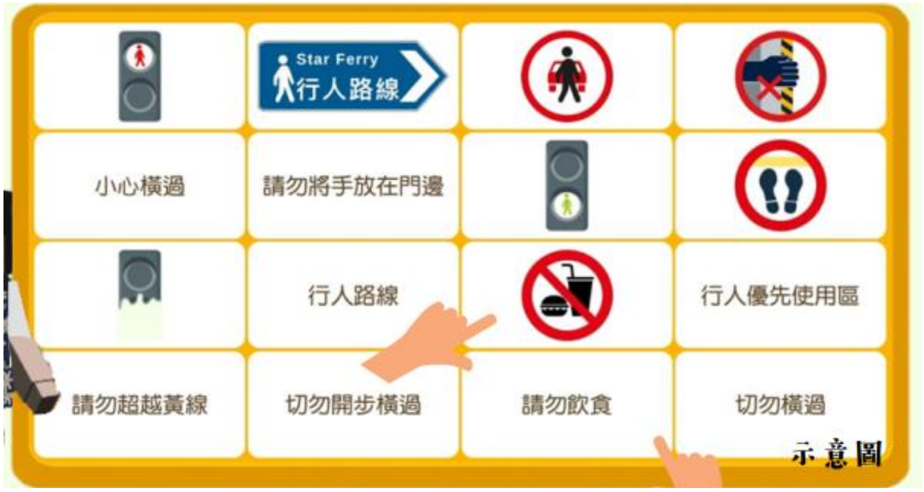

Figure S4. Safe City mobile game screenshot – Multiple Choice Questions

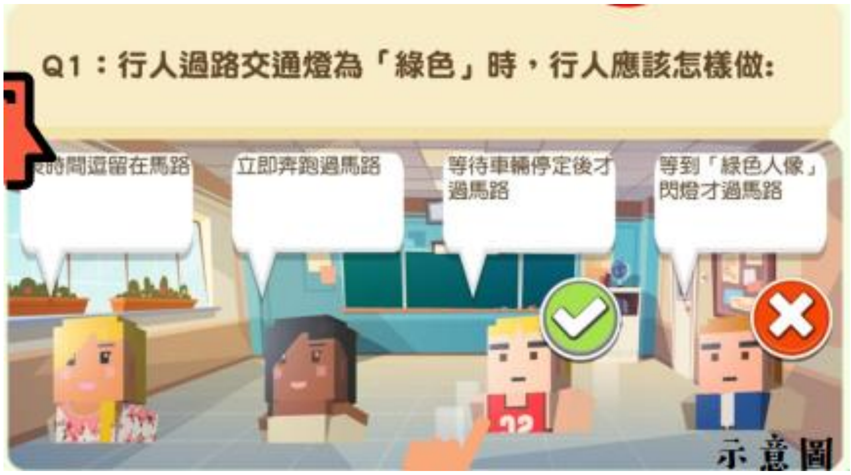

Figure S5. Safe City mobile game screenshot – Whac-a-Mole game

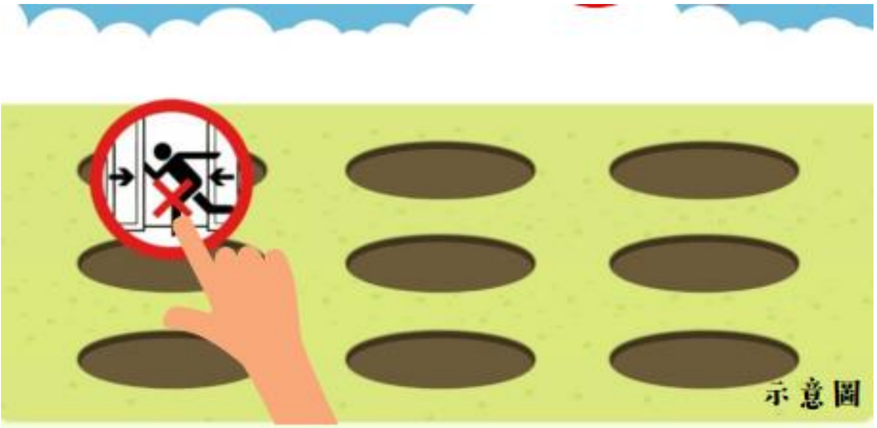

Figure S6. Generalized addictive models of safe knowledge (A), safe behavior (B), internalizing problem (C), and externalizing problem (D) improvement at the 1 month (T2) and 3 months post intervention (T3) as a function of cumulative game score.

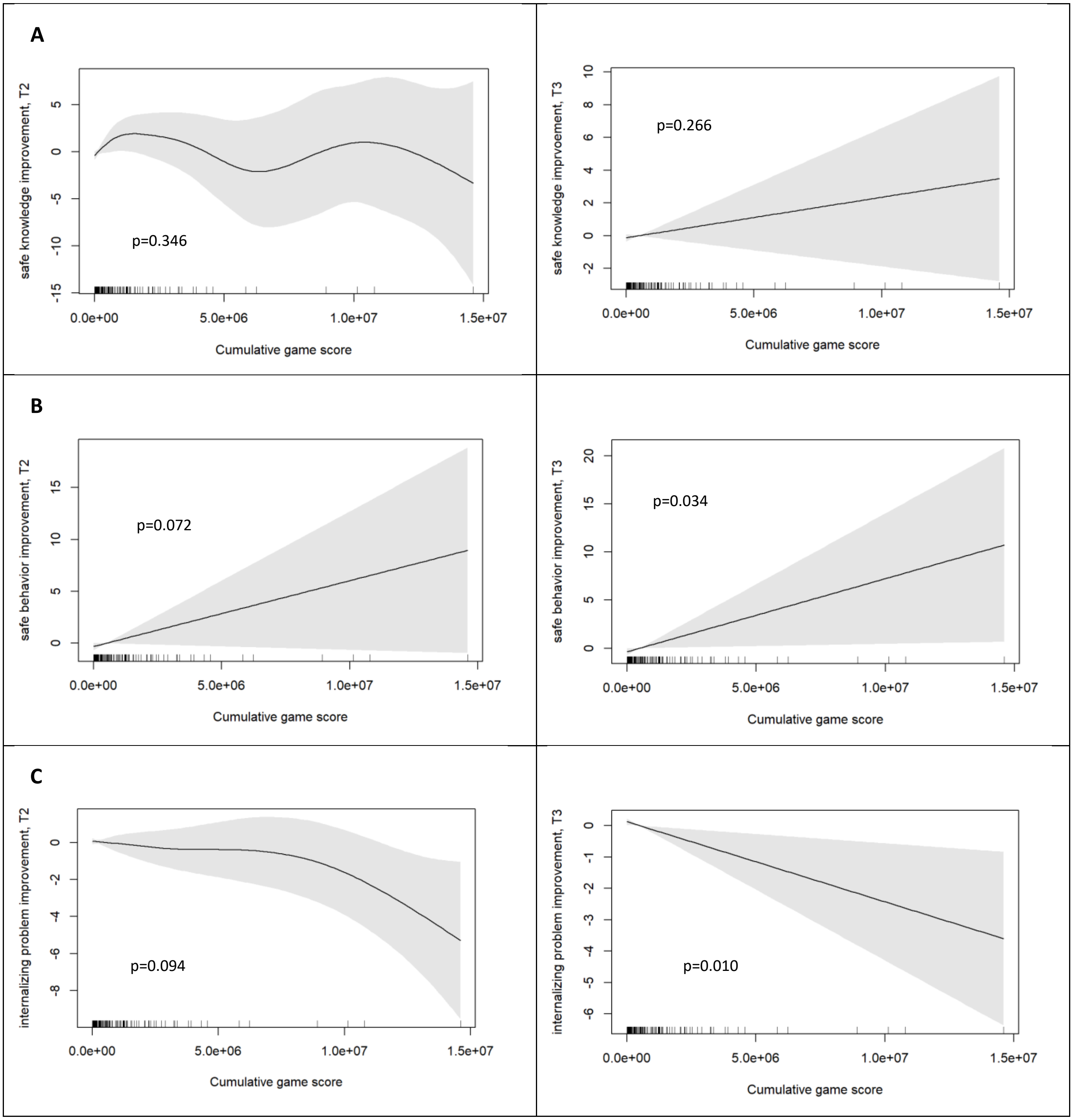

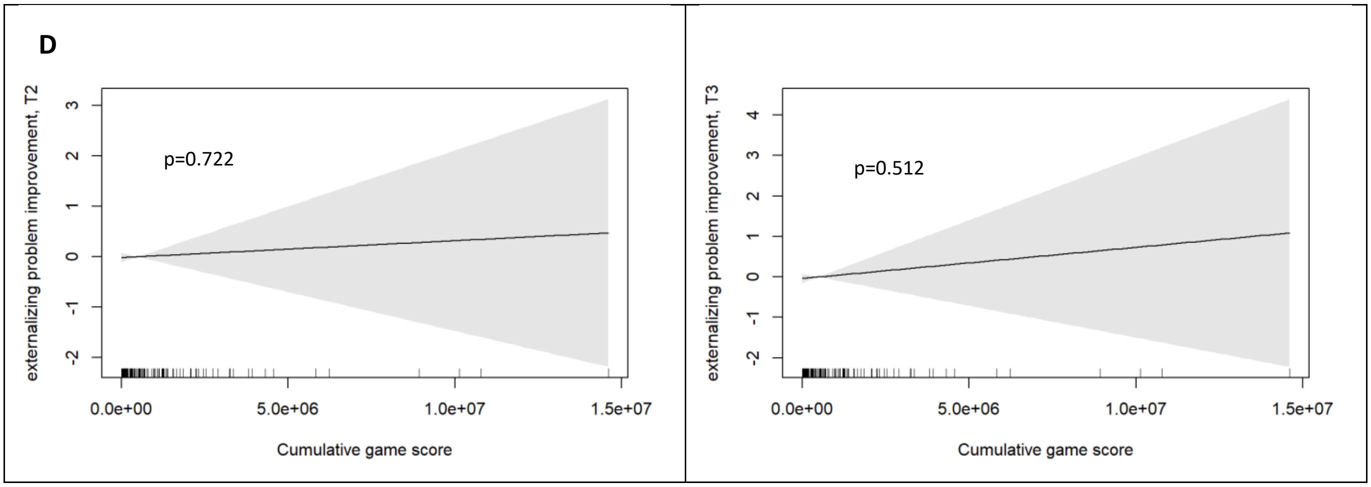

D
externalizing problem improvement, T2
p=0.722
Cumulative game score
0.0e+00
5.0e+06
1.0e+07
1.5e+07
externalizing problem improvement, T3
p=0.512
Cumulative game score

Figure S7. Generalized addictive models of safe knowledge (A), safe behavior (B), internalizing problem (C), and externalizing problem (D) improvement at the 1 month (T2) and 3 months post intervention (T3) as a function of the number of correct answers in multiple choice question (MCQ) mini games.

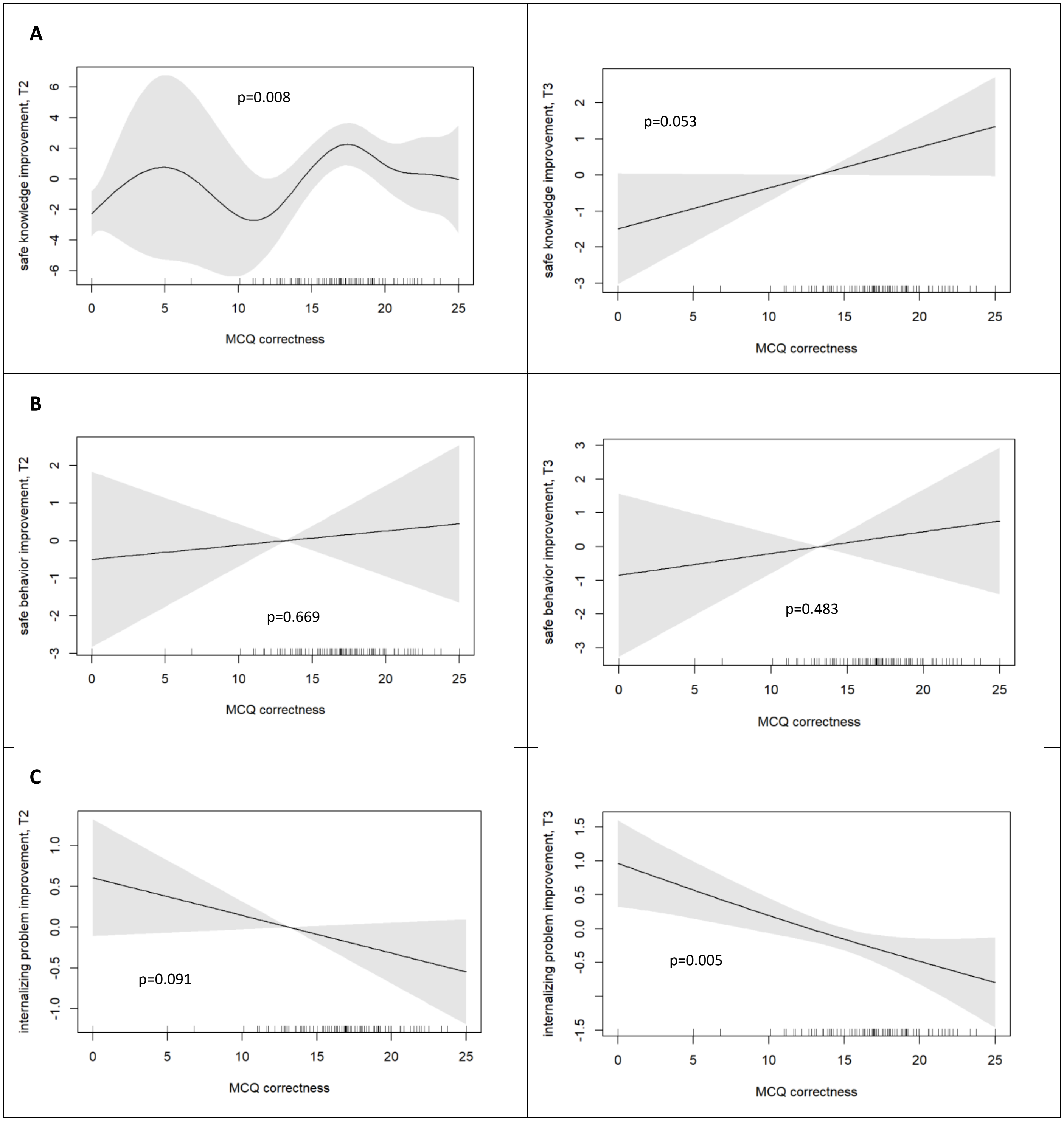

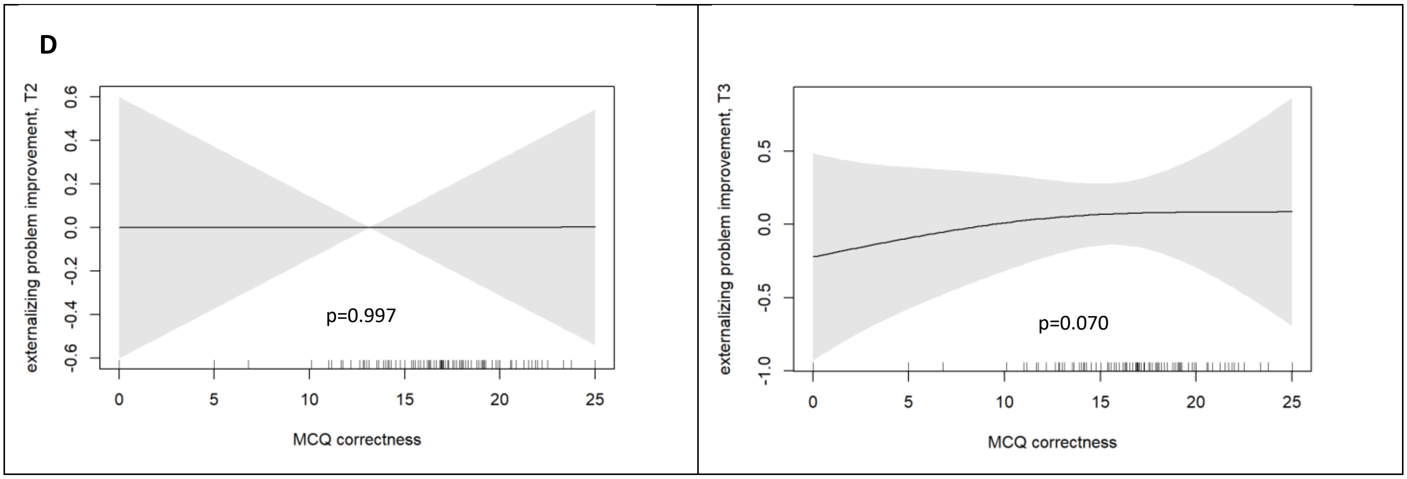
D
externalizing problem improvement, T2
MCQ correctness
p=0.997
externalizing problem improvement, T3
MCQ correctness
p=0.070

Figure S8. Generalized addictive models of safe knowledge (A), safe behavior (B), internalizing problem (C), and externalizing problem (D) improvement at the 1 month (T2) and 3 months post intervention (T3) as a function of the number of correct answers in spot the danger mini games.

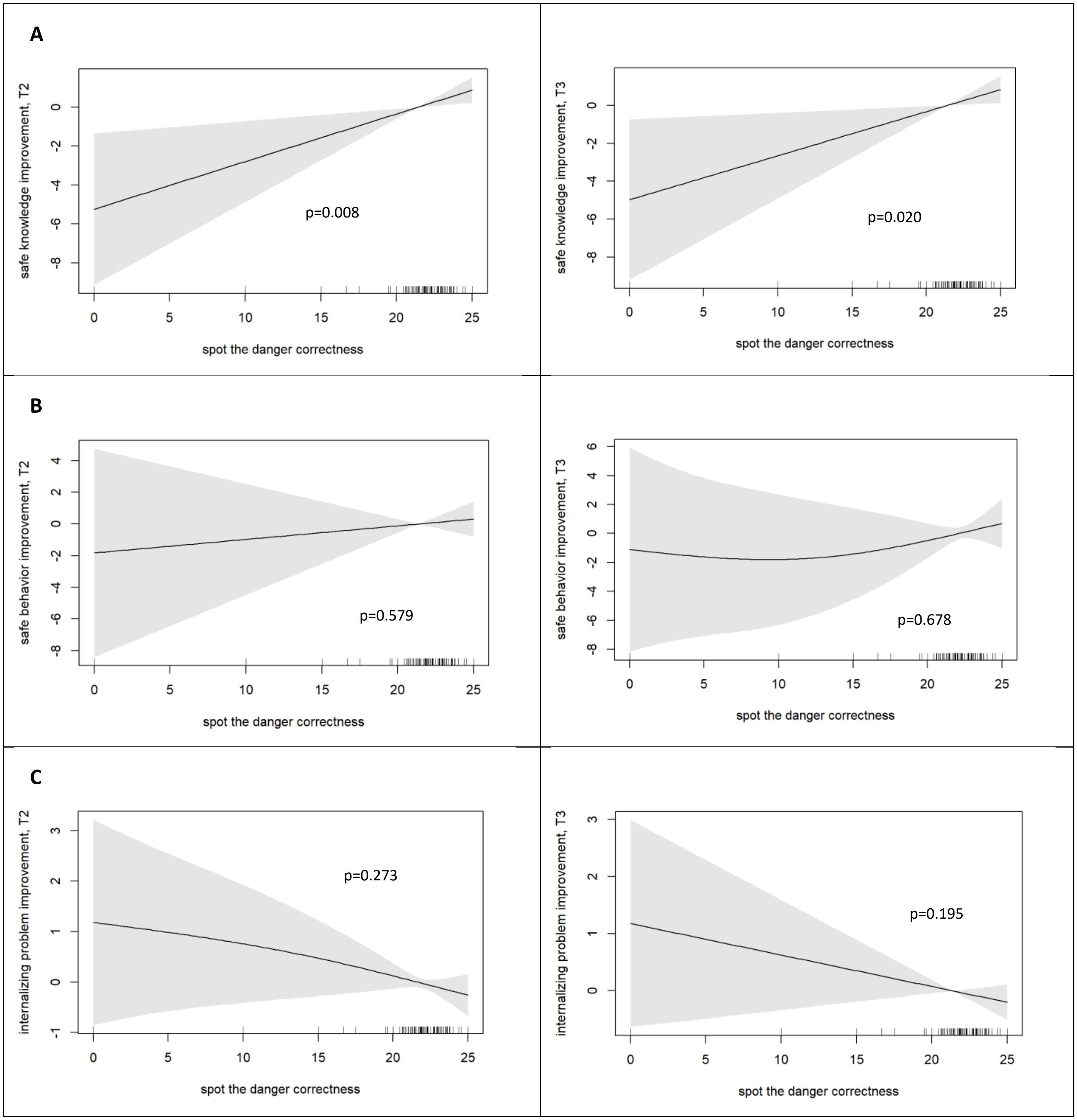

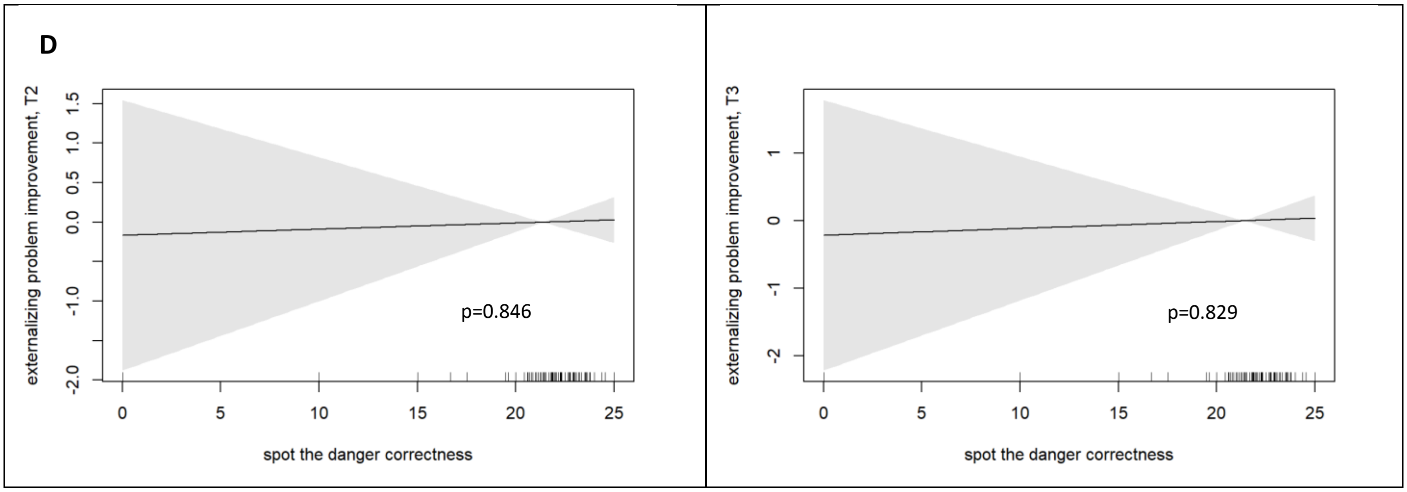
D
externalizing problem improvement, T2
-2.0
-1.0
0.0
0.5
1.0
1.5
p=0.846
0
5
10
15
20
25
spot the danger correctness
externalizing problem improvement, T3
-2
-1
0
1
p=0.829
0
5
10
15
20
25
spot the danger correctness

Figure S9. Generalized addictive models of safe knowledge (A), safe behavior (B), internalizing problem (C), and externalizing problem (D) improvement at the 1 month (T2) and 3 months post intervention (T3) as a function of the number of correct answers in matching mini games.

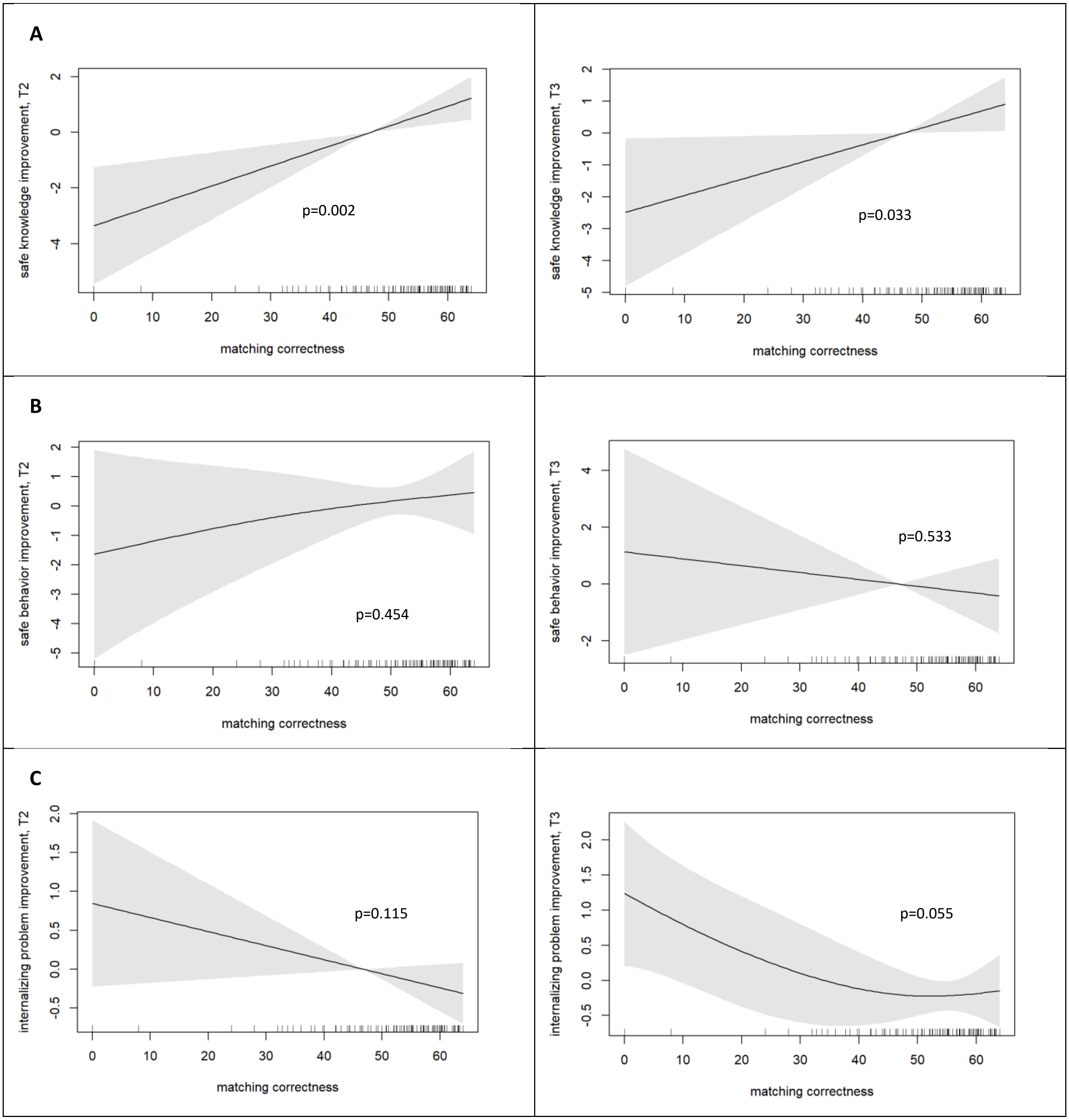

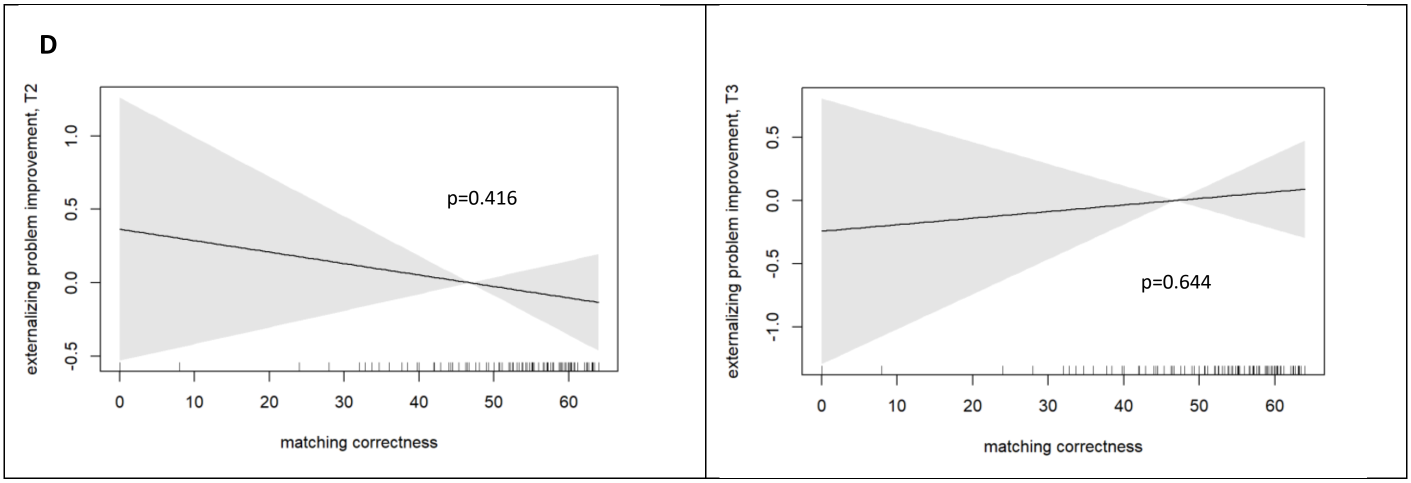

D
externalizing problem improvement, T2
p=0.416
matching correctness
externalizing problem improvement, T3
p=0.644
matching correctness
